# Intermittent pneumatic compression thromboprophylaxis among critically ill children: a single-center retrospective cohort study

**DOI:** 10.1016/j.rpth.2025.103222

**Published:** 2025-10-13

**Authors:** Nikhil Vallabhaneni, Marisol Betensky, A.J. Caberto, Neil A. Goldenberg, Anthony A. Sochet

**Affiliations:** 1 Department of Pediatrics, Division of Pediatric Critical Care Medicine, Medical College of Georgia, Augusta, GA, USA; 2 Department of Pediatrics, Johns Hopkins University School of Medicine, Baltimore, MD, USA; 3 Cancer and Blood Disorders Institute, Johns Hopkins All Children’s Hospital, St. Petersburg, FL, USA; 4 Data Coordinating Center for Pediatric Multicenter Studies, Johns Hopkins All Children’s Hospital, St. Petersburg, FL, USA; 5 Department of Medicine, Johns Hopkins University School of Medicine, Baltimore, MD, USA; 6 Institute for Clinical and Translational Research, Johns Hopkins All Children’s Hospital, St. Petersburg, FL, USA; 7 Department of Anesthesiology and Critical Care Medicine, Johns Hopkins University School of Medicine, Baltimore, MD, USA; 8 Division of Pediatric Critical Care Medicine, Department of Pediatrics, Johns Hopkins All Children’s Hospital, St. Petersburg, FL, USA

**Keywords:** hospital-acquired venous thromboembolism, mechanical thromboprophylaxis, pediatrics

## Abstract

**Background:**

Mechanical thromboprophylaxis, including intermittent pneumatic compression (IPC), is administered as primary prevention against hospital-acquired venous thromboembolism (HAVTE) in critically ill children without supportive evidence of VTE risk reduction.

**Objectives:**

We aimed to assess the association between IPC thromboprophylaxis and HAVTE in critically ill children.

**Methods:**

This was a single-center, retrospective cohort study of children aged <18 years hospitalized in a pediatric intensive care unit from 2020 to 2023. Those with a VTE present on admission or receiving therapeutic anticoagulation were excluded. Patients were classified into HAVTE risk tiers (low, moderate, and high) using previously validated criteria. The primary outcome was radiographically confirmed HAVTE. For each HAVTE risk tier, logistic regression was employed to test associations between HAVTE and IPC thromboprophylaxis, reporting odds ratios (ORs) with 95% CIs.

**Results:**

Of 4440 children studied, 30 (0.7%) had a HAVTE diagnosed at a median of 4.2 (IQR: 2.9-8.3) days after hospitalization. IPC was prescribed for 831 (18.7%) children. No association between IPC and HAVTE was detected for patients classified as high risk (OR: 0.27; 95% CI: 0.1-1.4) or moderate risk (OR: 0.81; 95% CI: 0.23-2.82) for HAVTE but was detected for those classified as low risk (OR: 6.3; 95% CI: 1.7-23.7).

**Conclusion:**

In this single-center cohort of critically ill children, IPC thromboprophylaxis was commonly prescribed but was not associated with HAVTE risk reduction among patients at high/moderate risk for HAVTE. These findings corroborate recent multicenter administrative database findings and underscore the need for risk-stratified clinical trials to evaluate the efficacy of IPC thromboprophylaxis in critically ill children.

## Essentials

• Critically ill children have increased risk of developing hospital-acquired venous thromboembolism (HAVTE).
• We sought to determine if intermittent pneumatic compression (IPC) prevents HAVTE in children.
• Among 4400 critically ill children, 18.7% were prescribed IPC and 0.7% developed HAVTE.
• Accounting for prothrombotic risk factors, IPC was not associated with reduced HAVTE frequency.

## Graphical abstract

**Figure:**
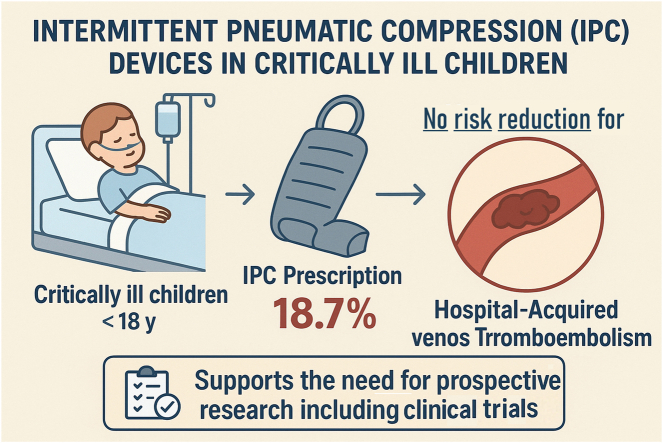

## 1 Introduction

Hospital-acquired venous thromboembolism (HAVTE), including deep venous thrombosis (DVT) and pulmonary embolism (PE), is associated with significant morbidity and mortality, affecting as many as 1 in 50 critically ill children [1,2]. Several pharmacologic thromboprophylaxis trials have been conducted in heterogenous pediatric subpopulations and clinical contexts, such as children with hematologic malignancies undergoing induction chemotherapy [3,4], acquired and congenital heart disease [5], COVID-19-related illness [6], and medically critical illness following insertion of a central venous catheter (CVC) [7,8]. Yet, in the absence of evidence of comparative efficacy derived from sufficiently powered phase 3 trials and along with concerns of attributable bleeding risk secondary to anticoagulation, there is substantial variability in VTE prevention practices across inpatient pediatric healthcare settings and populations [[9], [10], [11]].

As an alternative and/or as a supplement to pharmacologic thromboprophylaxis, mechanical thromboprophylaxis with intermittent pneumatic compression (IPC) devices is administered in as many as 12% to 24% encounters with critically ill adolescents [[10], [11], [12], [13]]. IPC may lower risk of HAVTE by optimizing venous blood flow via extremity venous compression and releasing endogenous endothelial anticoagulant mediators [[14], [15], [16]]. No clinical trials of IPC thromboprophylaxis have been conducted in children, and the presumption of its efficacy, coupled with the concern for bleeding from anticoagulant exposure, may result in suboptimal thromboprophylaxis [17]. The International Society on Thrombosis and Haemostasis (ISTH) Scientific and Standardization Committee Pediatric Thromboprophylaxis Working Party and the Children’s Healthcare Advancements in Thrombosis Consortium have stressed a need for research on optimal thromboprophylaxis strategies to inform practice guidelines [18]. Furthermore, at this time, there are no clinical practice guidelines from ISTH, the American Society of Hematology, and the Society for Critical Care Medicine recommending thromboprophylaxis of any type for critically ill children.

Although a recent multicenter administrative database study offered an updated estimate for mechanical thromboprophylaxis prescription among critically ill adolescents [13], limitations of that work (inherent to analyses derived from large administrative datasets) included incomplete details regarding IPC exposure, sequence of events, and salient prothrombotic factors. To address this gap, we performed a single-center, retrospective cohort study of critically ill children that included data collection of these variables. We sought to characterize IPC thromboprophylaxis use in this population and, after accounting for known prothrombotic risk factors, assess for independent association between IPC and HAVTE. Given the results of our prior administrative study, we hypothesized that there would be no independent association between IPC thromboprophylaxis and HAVTE, after accounting for established a priori HAVTE risk factors including CVC, prolonged hospitalization, and concurrent infection.

## 2 Methods

### 2.1 Study design and sampling criteria

We conducted a single-center, retrospective cohort study at a quaternary pediatric referral center, Johns Hopkins All Children’s Hospital, within a 28-bed pediatric intensive care unit (PICU). Inclusion criteria for the study were hospitalization in the PICU between January 1, 2020 and December 31, 2023 and age at admission <18 years. Exclusion criteria were the presence of a VTE on PICU admission or receipt of therapeutic anticoagulation. The study was reviewed and approved by the Johns Hopkins University Institutional Review Board (Approved: July 20, 2023; IRB: 00396103). The research described herein was carried out in accordance with the Code of Ethics of the World Medical Association (Declaration of Helsinki) for experiments involving humans and the the International Committee of Medical Journal Editors (ICMJE) Recommendations for the Conduct, Reporting, Editing and Publication of Scholarly Work in Medical Journals. The article follows STrengthening the Reporting of OBservational studies in Epidemiology (STROBE) reporting criteria for observational studies.

### 2.2 Data source

Potential subjects were screened using the Virtual Pediatric Systems data registry (VPS, LLC; http://www.myvps.org), in which institution-specific data are prospectively collected by data analysts at the institution. After applying the study criteria, all research data were extracted from an internal data warehouse via validated queries. Primary study outcomes, exposures, and HAVTE risk covariates (defined below) were reviewed and confirmed via manual electronic health record by 2 independent reviewers (N.V. and A.C.), with conflicting or discrepant data adjudicated by a third reviewer (A.S.).

### 2.3 Study definitions and outcomes

The primary outcome was radiographically confirmed HAVTE, including limb and neck DVT, PE, and organ-specific VTE (eg, cerebral venous sinus thrombosis). Radiographic confirmation included Doppler ultrasonography, computed tomography, venography, echocardiography, or magnetic resonance imaging. All HAVTEs were further characterized by the timing of diagnosis, potential relationship to a CVC, and degree of blood vessel occlusion (ie, complete vs partial occlusion). The primary exposure variable was IPC, including the timing of initiation and duration of exposure during PICU hospitalization. Throughout the study period, the IPC device was the Kendall-SCD-700 series (Cardinal Health). No patients were prescribed alternative mechanical thromboprophylaxis such as compression stockings.

Additional study variables collected for research included patient demographics, anthropometrics, prior VTE history, preexisting thrombophilia diagnoses (ie, antiphospholipid syndrome, antithrombin deficiency, protein C or S deficiency, factor V Leiden, prothrombin gene mutation, plasminogen activator inhibitor-1 mutation, and homocystinuria), prior or current malignancy diagnoses, acute treatment of infection and/or sepsis-related diagnoses, recent operative procedures (ie, within 5 days of hospitalization), acute mobility impairment (as measured by Braden Q mobility values [19]), presence of CVC (including peripherally inserted central catheters, percutaneous CVCs, and tunneled CVCs), acute invasive mechanical ventilation and duration thereof, chronic respiratory failure with tracheostomy, length of stay (LOS), ISTH-defined clinically relevant bleeding [20], and in-hospital mortality rates. Pharmacologic thromboprophylaxis defined as prescription of unfractionated heparin, low molecular weight heparin, or direct oral anticoagulants at prophylactic pediatric dosing regimens were noted for study.

### 2.4 Statistical analyses

To describe the study sample and cohorts defined by IPC thromboprophylaxis exposure and HAVTE events, summative statistics were used including proportions with percentages, means with SD, and medians with IQR depending on data type and distribution. Comparative statistics were employed including chi-squared and Fisher’s exact test for categorical data and Student’s *t*-test or Wilcoxon rank sum test for continuous data. To evaluate potential relationships with HAVTE for IPC and salient covariates, the HAVTE risk score for critically ill children developed by Arlikar et al. [21] and subsequently validated by Mahajerin et al. [22] was used. The score applies relative weighting of salient clinical features (ie, CVC presence, LOS ≥4 days, and hospitalization for sepsis/infection) to generate VTE risk tiers (ie, low, moderate, and high VTE risk). Within each risk tier, unadjusted logistic regression was employed to assess for potential association between IPC thromboprophylaxis and HAVTE, yielding odds ratios (ORs) with corresponding Wald 95% CIs. Given the anticipated low HAVTE event rate, no plan was made for the creation of an adjusted model per se. However, by applying the risk-stratification tiers, adjustment for salient HAVTE risk factors was indirectly achieved. Missing data were not imputed. Each PICU encounter was considered independent. All tests were 2-sided, and type I error was set at 0.05. All analyses were completed using Stata v15.1 software (Stata Corporation).

## 3 Results

### 3.1 IPC thromboprophylaxis frequency and characteristics

**Figure 1:**
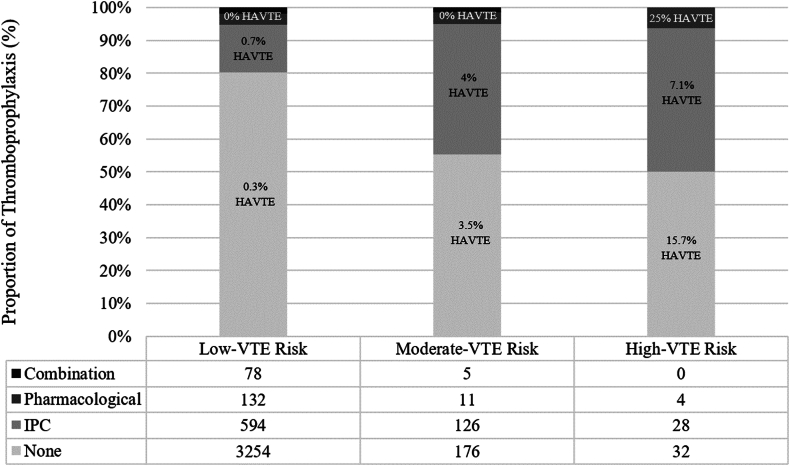
Types of thromboprophylaxis administered across tiers of low, moderate, and high risk for hospital-acquired venous thromboembolism in the study population. HAVTE, hospital-acquired venous thromboembolism; IPC, intermittent pneumatic compression; VTE, venous thromboembolism.

General population characteristics for the study sample and cohorts defined by the exposure to IPC are listed in Table 1. A total of 4400 children met the criteria for the study, of which 831 (18.7%) were prescribed IPC at a median of 1.2 (IQR: 0.5-9.6) hours after admission. The median duration of IPC thromboprophylaxis exposure was 3.8 (IQR: 1.7-7.9) days. A total of 185 (4.2%) children received pharmacologic thromboprophylaxis alone (all of whom received low molecular weight heparin), and 45 (1%) children received both pharmacologic and IPC (Figure 1).

**Table 1:** Clinical features and outcomes for critically ill children with and without exposure to IPC thromboprophylaxis.

Variables	Overall study sample *N* = 4400	No IPC *n* = 3609	IPC *n* = 831	*P*
Age, y, median (IQR)	5 (1-13)	4 (1-13)	11 (3-15)	<.001
Weight, kg, median (IQR)	21 (11.4-48.7)	16.6 (10.5-39)	50 (32-67)	<.001
Body mass index, kg/m^2^, median (IQR)	18.1 (15.9-21.4)	17.6 (15.6-20.6)	20.8 (17.8-26.8)	<.001
Prior history of VTE, *n* (%)	124 (2.8)	88 (2.4)	36 (4.3)	.005
Prior thrombophilia diagnosis, *n* (%)	17 (0.4)	10 (0.3)	7 (0.8)	.027
Prior malignancy, *n* (%)	99 (2.2)	70 (1.9)	29 (3.5)	.009
Current malignancy, *n* (%)	100 (2.3)	67 (1.9)	33 (3.9)	.001
Current infection and/or sepsis, *n* (%)	475 (10.7)	325 (9)	150 (18.1)	<.001
Operative procedure ≤5 d, *n* (%)	935 (21.1)	744 (20.6)	191 (23)	.131
Pharmacologic TP, *n* (%)	230 (5.2)	185 (5.1)	45 (5.4)	.729
Initiated timing, days, median (IQR)	0.4 (0.2-1.2)	0.4 (0.2-1.3)	0.4 (0.1-0.9)	.322
Braden Q mobility on admission, *n* (%)				<.001
No limitation	2,368 (53.7)	2,039 (56.9)	329 (39.7)	
Partial or some limitation	1,771 (40.2)	1,389 (38.8)	382 (46.1)	
Complete limitation	272 (6.2)	155 (4.3)	117 (14.1)	
Any bleeding event, *n (%)*	78 (1.8)	51 (1.4)	27 (3.3)	.001
Central venous catheterization, *n* (%)	223 (5)	137 (3.8)	86 (10.4)	<.001
Chronic tunneled catheter, *n* (%)	40 (0.9)	29 (0.8)	11 (1.3)	.155
Invasive mechanical ventilation, *n* (%)	419 (9.4)	277 (7.7)	142 (17.1)	<.001
Duration, days, median (IQR)	3.7 (1.3-11.9)	3.6 (1.1-11.9)	4.1 (1.9-11)	.146
Tracheostomy dependence, *n* (%)	160 (3.6)	123 (3.4)	37 (4.5)	.149
Blood product transfusion, *n* (%)				
Fresh frozen plasma	66 (1.5)	40 (1.1)	26 (3.1)	<.001
Red blood cells	278 (6.3)	203 (5.6)	75 (9)	<.001
Platelets	103 (2.3)	67 (1.9)	3 (4.3)	<.001
In-hospital mortality, *n* (%)	69 (1.6)	44 (1.2)	25 (3)	<.001
Length of stay, median (IQR)				
Pediatric intensive care unit	1.6 (0.9-3.3)	1.5 (0.9-2.8)	2.6 (1.1-6.2)	<.001
Hospital	3.4 (1.9-6.8)	3.2 (1.8-5.9)	5 (2.9-11.9)	<.001

IPC, intermittent pneumatic compression; TP, thromboprophylaxis; VTE, venous thromboembolism.

Patients who received IPC, as compared to those who did not, were older (median age: 11 [IQR: 3-15] vs 4 [IQR: 1-13] years), had greater body mass index (median 20.8 [IQR: 17.8-26.8] vs 17.6 [IQR: 15.6-20.6] kg/m^2^), and more frequently had impaired mobility at the time of PICU admission (60.2% vs 43.1%) (all *p* < 0.001). The prevalence of CVC (10.4% vs 3.8%) and invasive mechanical ventilation (17.1% vs. 7.7%) were greater among children who did, vs did not, receive IPC, as was in-hospital mortality (3% vs 1.2%) (all *P* < .001). No complications related to IPC were documented.

### 3.2 HAVTE and clinically relevant bleeding frequencies and characteristics

A total of 30 (0.7%) children developed a HAVTE with a median time to diagnosis of 4.2 (IQR: 2.9-8.3) days after PICU admission. The majority of HAVTE were DVT (29 of 30 cases) and a single case of PE. For DVT, 51.7% were identified in veins of the upper extremity and neck and the remaining 48.3% in veins of the lower extremity and abdomen. General characteristics of patients with and without subsequent HAVTE diagnosis are shown in Table 2. Compared with children who did not develop a HAVTE, those who did more frequently had a history of prior VTE (30% vs 2.6%), an acute infection or sepsis-related diagnosis (43.3% vs 10.5%), impaired mobility at admission (73.3% vs 46.1%), a CVC (60% vs 4.7%), and acute invasive mechanical ventilation (66.7% vs 9.1%) (all *P <* .001). Those with, as compared to those without, HAVTE experienced a longer median PICU LOS (12.4 [IQR: 8.3-20.5] vs 1.6 [IQR: 0.9-3.2] days, *P* < .001) and higher inpatient mortality (10% vs 1.5%, *P* = .011).

**Table 2:** Clinical features and outcomes for critically ill children with and without development of a hospital-acquired venous thromboembolism.

Variables	Overall sample *N* = 4440	No HAVTE *n* = 4410	HAVTE *n* = 30	*P*
Age, y, median (IQR)	5 (1-13)	5 (1-13)	3.5 (1-7)	.098
Weight, kg, median (IQR)	21 (11.4-48.7)	21 (11.5-48.9)	11.4 (6.5-23.5)	.006
Body mass index, kg/m^2^, median (IQR)	18.1 (15.9-21.4)	18.1 (15.9-21.4)	19.3 (15.6-28.9)	.424
Prior history of VTE, *n* (%)	124 (2.8)	115 (2.6)	9 (30)	<.001
Prior thrombophilia diagnosis, *n* (%)	17 (0.4)	17 (0.4)	0 (0)	>.999
Prior malignancy, *n* (%)	99 (2.2)	97 (2.2)	2 (6.7)	.143
Current malignancy, *n* (%)	100 (2.3)	98 (2.2)	2 (6.7)	.146
Current infection and/or sepsis, *n* (%)	475 (10.7)	462 (10.5)	13 (43.3)	<.001
Operative procedure ≤5 d, *n (%)*	935 (21.1)	931 (21.1)	4 (13.3)	.374
IPC thromboprophylaxis, *n* (%)	831 (18.7)	820 (18.6)	11 (36.7)	.018
Initiated timing, days, median (IQR)	0.05 (0.02-0.4)	0.02 (0.05-0.4)	0.02 (0.01-0.05)	
Duration of exposure, median (IQR)	3.8 (1.7-7.9)	3.6 (1.7-7.6)	11.1 (5.6-25.3)	
Pharmacologic thromboprophylaxis, *n (%)*	230 (5.2)	229 (5.2)	1 (3.3)	.647
Any type of TP, *n (%)*	980 (22.1)	975 (22.1)	5 (16.7)	.474
Both TP concurrent, *n (%)*	83 (1.9)	83 (1.9)	0 (0)	.448
Braden Q mobility on admission, median (IQR)	0 (0-1)	0 (0-1)	1 (0-1)	<.001
No limitation, *n* (%)	2368 (53.7)	2360 (53.9)	8 (26.7)	
Some limited, *n* (%)	1771 (40.2)	1756 (40.1)	15 (50)	
Completed limited, *n* (%)	272 (6.2)	265 (6)	7 (23.3)	<.001
Any bleeding event, *n* (%)	78 (1.8)	75 (1.7)	3 (10)	.001
Central venous catheter, *n* (%)	223 (5)	205 (4.7)	18 (60)	<.001
Chronic tunneled line, *n* (%)	40 (0.9)	39 (0.9)	1 (3.3)	.238
Invasive mechanical ventilation, *n* (%)	419 (9.4)	399 (9.1)	20 (66.7)	<.001
Duration, days, median (IQR)	3.7 (1.3-11.9)	3.5 (1.2-11.9)	8.1 (5-19.3)	.009
Tracheostomy dependence, *n* (%)	160 (3.6)	158 (3.6)	2 (6.7)	.295
In-hospital mortality, *n* (%)	69 (1.6)	66 (1.5)	3 (10)	.011
Length of stay, median (IQR)				
Pediatric intensive care unit	1.6 (0.9-3.3)	1.6 (0.9-3.2)	12.4 (8.3-20.5)	<.001
Hospital	3.4 (1.9-6.8)	3.4 (1.9-6.6)	21.6 (14.1-29.6)	<.001
VTE risk category, *n* (%)				<.001
Low	4058 (91.4)	4049 (91.8)	9 (30)	
Moderate	318 (7.2)	307 (7)	11 (36.7)	
High	64 (1.2)	54 (1.2)	10 (33.3)	

HAVTE, hospital-acquired venous thromboembolism; TP, thromboprophylaxis; VTE, venous thromboembolism.

A total of 78 (1.8%) children developed a clinically relevant bleeding event during hospitalization including 41 (52.6%) with gastrointestinal bleeding, 36 (46.2%) pulmonary hemorrhage, and 11 (14.1%) intracranial hemorrhage. Of the 255 children who received pharmacologic thromboprophylaxis, 5 (2.2%) developed a clinically relevant bleed, and this rate was not detectably different from those who did not receive pharmacologic thromboprophylaxis (1.7%, *P* = .621). No differences in rates of clinically relevant bleeding existed for those children with and without surgical intervention during hospitalization (2.1% vs 2.6%, *P* = 0.842).

### 3.3 Association between HAVTE risk and use of IPC thromboprophylaxis

**Figure 2:**
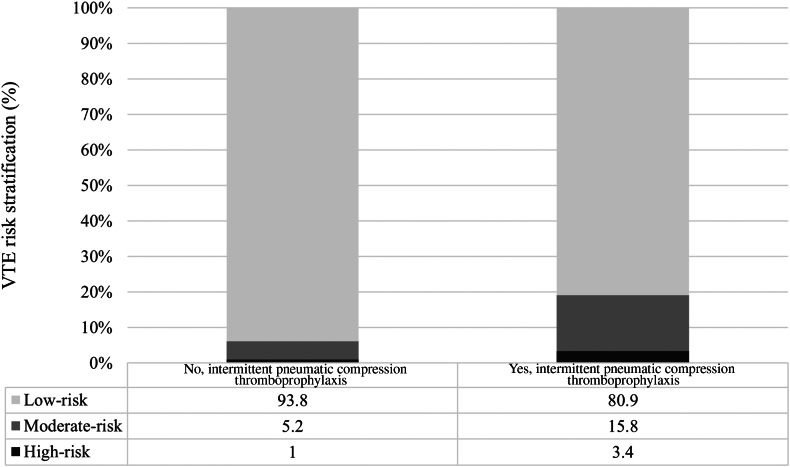
Venous thromboembolism risk stratification by groups defined by ITP thromboprophylaxis exposure in the study population. VTE, venous thromboembolism.

**Figure 3:**
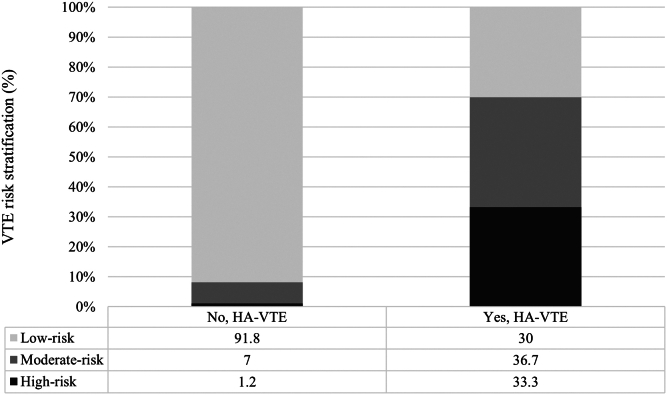
Venous thromboembolism risk stratification by groups defined by development of a hospital-acquired venous thromboembolism among critically ill children. HAVTE, hospital-acquired venous thromboembolism; VTE, venous thromboembolism.

Figure 2 depicts categorization of VTE risk for those who did and did not receive IPC and Figure 3 by those who did and did not subsequently develop a HAVTE. Overall, 64 children (1.4%) were categorized with high risk and 318 (7.2%) with moderate risk for HAVTE. Those classified as high risk for HAVTE were more frequently prescribed IPC thromboprophylaxis (3.4% vs 1%, *P* < .001). Similarly, those classified as high risk for VTE more frequently went on to develop a HAVTE (33.3% vs 1.2%, *P* < .001). Unadjusted logistic regression for HAVTE occurrence did not reveal an association with use of IPC among those classified with high risk (OR: 0.27; 95% CI: 0.1-1.4; *P* = .117) and moderate risk (OR: 0.81; 95% CI: 0.23-2.82; *P* = .741) for HAVTE. For those classified with low risk, a positive association between use of IPC and HAVTE occurrence was detected (OR: 6.3; 95% CI: 1.7-23.7).

## 4 Discussion

In this single-center retrospective cohort study of critically ill children, we observed the rate of IPC thromboprophylaxis administration to be 18.7% and did not detect an associated risk reduction in HAVTE, even after accounting for moderate and high risk using validated pediatric HAVTE risk tiers based upon salient prothrombotic risk factors including CVC, prolonged LOS, and a concurrent infection. In the low-risk tier (those children without a CVC), there was an increased risk of HAVTE observed, but it was likely confounded by the low event rate in this subgroup (0.2%). Prior work using a multicenter administrative dataset estimated the rate of all types of mechanical thromboprophylaxis of 19.6% for a cohort of over 100,000 critically ill adolescents [13]. In that study, we similarly found no association between mechanical thromboprophylaxis with HAVTE development. The present study corroborates those findings and addresses several key limitations from the prior work with regard to the capacity to authenticate the sequence of events and confirm exposure to IPC exposure prior to HAVTE development. A number of factors could have contributed to the absence of risk reduction noted in our dataset and include patient demographic and illness context heterogeneity. For example, older children were noted to be prescribed IPC more often than younger children, who are not developmentally equivalent and frequently may be hospitalized for heterogenous conditions. In a sensitivity analysis (not shown), the inclusion of age as a potential covariate in all risk tiers did not alter our study results. Although these findings require further evaluation via a prospective cohort design, they infer the efficacy of IPC as a primary method for VTE prevention in critically ill children may be insufficient and warrants prospective investigation via clinical trials.

Data in support of IPC as thromboprophylaxis are derived from adult trial populations of predominantly surgical patients [23]. In a meta-analysis of adult trials, adjunctive IPC (ie, combined with pharmacologic thromboprophylaxis) was associated with a decreased risk of any HAVTE (relative risk [RR]: 0.53; 95% CI: 0.35-0.81) and specifically DVT (RR: 0.52; 95% CI: 0.33-0.81), but not PE (RR: 0.73; 95% CI: 0.32-1.68) [24]. To date, no phase 3 pediatric clinical trials of thromboprophylaxis, including those using mechanical or pharmacologic strategies assessed independently or in combination, have been conducted. As a result, only observational data have been reported in children and are in contrast to adult trial findings. Due to insufficient evidence that mechanical thromboprophylaxis reduces HAVTE risk in children, its application in lieu of pharmacologic thromboprophylaxis may be insufficient. Our study sought to characterize the duration and timing of IPC thromboprophylaxis and observed a median time to prescription of IPC of 1.2 hours of PICU admission. However, we cannot confirm retrospectively that the absence of IPC efficacy in VTE risk reduction in our study population was at least partially accounted for by compliance with IPC orders (ie, whether a child was wearing the device).

While the risk clinically relevant bleeding among critically ill children is elevated compared to other pediatric populations [25,26], recent meta-analyses suggest that attributable bleeding risk from low molecular weight heparin thromboprophylaxis is low in children, between 0.6% and 2.3% [27,28]. An increasing body of phase 2 pediatric trial-derived evidence similarly conveys the safety and efficacy of pharmacologic thromboprophylaxis across a number of heterogenous contexts but does not concurrently report on adjunctive mechanical thromboprophylaxis including IPC. Our data from this study corroborates these reports in that the rates of clinically relevant bleeding were not observably different for those who did and did not receive pharmacologic thromboprophylaxis. Although nearly 20% of our overall study population and 50% of those identified with high VTE risk were prescribed some type of thromboprophylaxis, we speculate that provider perceptions of bleeding risk and the absence of efficacy data preclude the common application of these preventative strategies in children. One consideration for the planning and conduct of pediatric trials would be studying pediatric populations presumed at high bleeding risk such as those immediately following major trauma or in the postoperative period who could not be randomized to receive anticoagulants.

### 4.1 Limitations

While this study did include >4000 pediatric encounters, the aggregate HAVTE rate was low and did not permit the inclusion of covariates in a multivariable logistic regression model. As such, a number of confounding factors could have influenced our ability to detect an association between IPC use and HAVTE. However, by stratifying children using established risk tiers, the models inherently account for the presence of a CVC, prolonged hospital LOS, and inflammatory/infectious processes. Given that very few children received combined pharmacologic and IPC thromboprophylaxis, we were unable to determine the additive effect of multiple VTE preventative interventions in our study. Due to the small number of HAVTEs identified, it was not possible to distinguish associations between IPC and location of DVT (upper vs lower extremity). Although we could determine the timing and total duration of IPC thromboprophylaxis prescription, its actual exposure (ie, the amount of time it remained on the patients while ordered) cannot be confirmed retrospectively. Therefore, the absence of an association may be related to inadequate exposure, which could be addressed in a future prospective investigation.

## 5 Conclusion

In this single-center retrospective observational cohort study, we found no risk reduction in HAVTE in association with the use of IPC thromboprophylaxis in critically ill children, after adjustment for a priori risk of HAVTE including presence of a CVC, duration of hospitalization, and concurrent infectious processes. Future risk-stratified clinical trials of IPC thromboprophylaxis are needed to further evaluate the potential efficacy of this VTE preventative strategy among critically ill children.
